# Type 2 Autoimmune Hepatitis in a Male Patient with a Rare Complication of Vasculitis

**DOI:** 10.7759/cureus.7354

**Published:** 2020-03-21

**Authors:** Ashar Shahid, Avinash Punshi, Bilal Ahmed Khan, Maaz Bin Nazir, Hidayat Ullah

**Affiliations:** 1 Internal Medicine, Dow University of Health Sciences (DUHS), Karachi, PAK; 2 Internal Medicine, Civil Hospital Karachi, Karachi, PAK; 3 Internal Medicine, Dow Medical College, Dow University of Health Sciences (DUHS), Karachi, PAK; 4 Medicine, Dow Medical College, Dow University of Health Sciences (DUHS), Karachi, PAK

**Keywords:** vasculitis, auto immune hepatitis, cirrhosis, interface hepatitis

## Abstract

Autoimmune hepatitis (AIH) is chronic inflammation of hepatocytes due to immune cells attacking the patient’s own hepatocytes, histologically characterized by interface hepatitis. The disease can be serious, and if left untreated, it can lead to cirrhosis of the liver and eventual liver failure. It occurs more frequently in females. The standard treatment for AIH includes corticosteroids. There are two main treatment regimens, which include either prednisolone alone or prednisone and azathioprine. Although, liver transplantation is certainly the treatment of choice, it has not yet been established on a large scale worldwide. We present here the case of a 22-year-old male, with autoimmune hepatitis and unspecified vasculitis.

## Introduction

Autoimmune liver disorders are a group of immune-mediated hepatic disorders, which include autoimmune hepatitis (AIH), primary biliary cirrhosis (PBC) and primary sclerosing cholangitis (PSC) [1]. Patients can present with a range of signs and symptoms depending on the stage of the disease, with hepatomegaly, ascites, dark urine and pale stools being the most common. In addition to these, jaundice, itching, fatigue, nausea, vomiting, joint pain and abdominal discomfort can also be seen in patients. There are two types of autoimmune hepatitis: type 1 is characterized by increased levels of anti-nuclear antibodies (ANA) and anti-smooth muscle antibodies (ASMA); and type 2 autoimmune hepatitis is characterized by increased levels of anti-liver/kidney microsomal antibodies (anti-LKM) and anti-liver cytosol 1 (ALC 1) antibodies. Type 1 occurs mostly in young girls.

Autoimmune hepatitis is characterized by increased aminotransferases, immunoglobulins and alkaline phosphatase (ALP) levels. Regulatory T cells (Tregs) extracted from children and adults with AIH have been found to be largely non-functional, signifying that Treg scarcity is involved in the pathogenesis of AIH [2].

Frequency data from Western Europe vary from 0.8 to 3 per 100,000, with an occurrence varying from 11 to 24 per 100,000 [3]. In Asia, AIH appears to be less common, with frequency statistics varying between 0.08 and 0.15 in Japan [4].

We present here the case of a male patient with autoimmune hepatitis and unspecified vasculitis.

## Case presentation

A 22-year-old male arrived in the emergency room with blackening of both hands and feet. According to the patient, he initially felt numbness in his fingers and toes, and blackening occurred overnight in all four limbs. He also felt weakening and pain in his muscles and complained of high-grade fever associated with rigors and chills. He had non-bloody vomiting, which contained almost everything that he had eaten or drunk. He had decreased appetite and weight loss. The patient had an accident a year previously, as a result of which he sustained fractures in his right thigh and foot. The patient did not undergo any orthopedic surgery, contrary to medical advice. The patient had a positive family history, and both his parents were hepatitis-C positive.

A general physical examination revealed the following: mild anemia, no jaundice, tachycardia (98 beats/min), low blood pressure (100/60 mmHg) and a high-grade fever (102°F). An abdominal exam showed hepatomegaly, with mild tenderness in the right upper quadrant. Cardiovascular and central nervous system (CNS) examinations were unremarkable. Complete blood count (CBC) revealed mild anemia (11.6 g/dl), lymphocytic leukocytosis (99), raised erythrocyte sedimentation rate (ESR) (93) and decreased platelets (56 × 10^3^/ul). Serum protein electrophoresis showed decreased albumin (2.2 g/dl), increased globulin (6.2 g/dl) and an albumin-globulin ratio of 0.35. The patient had normal bilirubin levels, with increased alanine transaminase (ALT) (75 U/L) and increased ALP (298 U/L). His D-dimer level was markedly increased (>15000), but a Doppler ultrasound of both the upper and lower limbs was normal. Both p-ANCA and c-ANCA levels were normal. Hepatitis B surface antigen (HBsAg) and anti-hepatitis C virus (HCV) were present, and both came out negative. The patient tested positive for anti-mitochondrial antibodies. Type 1 and type 2 autoimmune hepatitis antibodies were sent to the lab. Anti-liver cytosol antibodies tested positive, showing positive results for type 2 autoimmune hepatitis. The patient had elevated levels of D-dimer and decreased platelets, but the Doppler study of both upper and lower limbs was normal.

**Figure 1:**
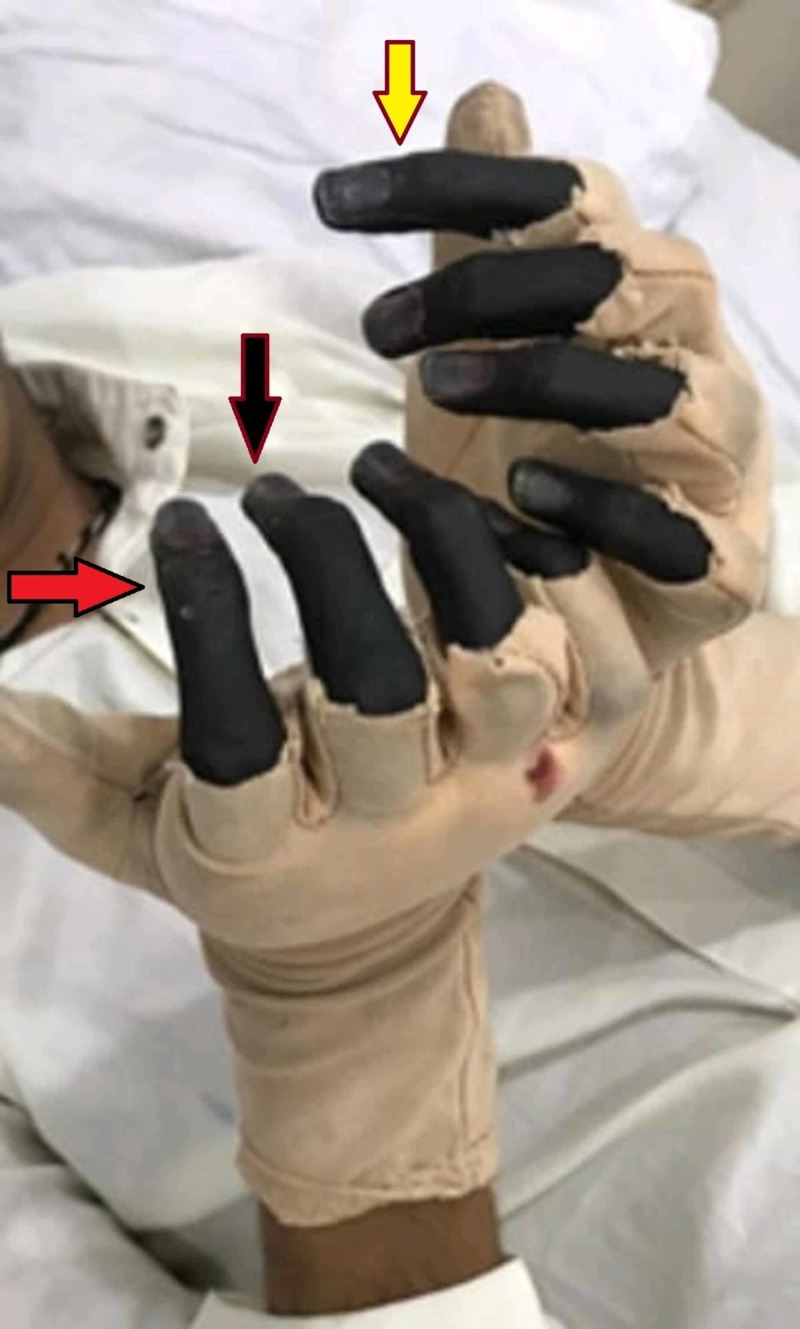
Dry gangrene of patient’s hand

The patient was given nifedipine for the vasculitis and steroids for the autoimmune hepatitis. He remained in the medical ward for two months, during which time all this workup was completed, and the patient was discharged. He presented again two weeks later with an infection in both hands (dry gangrene) (Figure 1). We referred him to the orthopedic surgery department for amputation of both hands. On the advice of the orthopedic consultant, the patient was sent to vascular surgery, and his amputation was planned.

The patient gave his informed consent to the probable publication of this case report. To preserve his privacy, we have not indicated any details that might give a clue as to his identity, and all information has been provided anonymously.

## Discussion

Women are inflicted with this disorder more than men. For type 1 AIH, the female to male ratio is 4:1, and for type 2, the ratio is 10:1 [5, 6]. An association between autoimmune liver disease and other autoimmune diseases such as Sjögren syndrome, systemic sclerosis and rheumatoid arthritis is often reported. In contrast, the association between vasculitis and autoimmune hepatitis is reported less frequently. AIH mainly affects children and young adults, and it presents more frequently as end-stage cirrhosis of the liver [7]. Anti-mitochondrial antibodies are usually absent in autoimmune hepatitis. If they are present, a diagnosis of primary biliary cirrhosis or an overlap syndrome should be considered, including both primary biliary cirrhosis and autoimmune hepatitis.

A study of children with autoimmune hepatitis recorded the following clinical findings and their prevalence: jaundice (58%), non-specific weakness (57%), anorexia (47%), abdominal pain (38%) and pallor (26%) [8]. Similar findings were present in our case.

Type 2 autoimmune hepatitis is more common in children and young adults, who usually present with an acute onset of AIH, just like in our case. Our patient also tested positive for anti-mitochondrial antibody 2, hence our diagnosis of AIH associated with primary biliary cirrhosis, but in the liver biopsy, chronic inflammation with infiltration of lymphocytes was seen.

Small-vessel vasculitis is mostly present in primary biliary cirrhosis rather than AIH. The reason for the relation between AIH and small-vessel vasculitis is not yet known [9]. However, HLA: A1, B8 and DR3 mutations are associated with both PSC and AIH vasculitis in patients [10, 11]. The same genetic predisposition is found in Churg-Strauss syndrome [12]. Wegener’s granulomatosis, microscopic polyangiitis and Churg-Strauss syndrome are forms of ANCA-associated vasculitis, but in our patient, serum p-ANCA and c-ANCA were both negative, although he presented with signs of vasculitis. His lungs, nasopharynx and kidneys were also not affected.

The standard treatment for AIH includes corticosteroids, which is based on the results of a clinical trial that took place in 1970 [13]. There are two main treatment regimens, which include either prednisolone alone or prednisone and azathioprine [14]. About 9% of cases become unresponsive to these regimens, so other immunosuppression drugs are being evaluated, such as cyclophosphamide, tacrolimus and mycophenolate mofetil [15,16]. The American Association for the Study of Liver Diseases (AASLD) and the British Society of Gastroenterology (BSG) recommend combination therapy, using the corticosteroid prednisone with azathioprine. Although liver transplantation is helpful in AIH, it has not yet been established on a large scale worldwide. However, AIH accounts for about 2.6% of liver transplants in Europe and about 5.9% in the USA [17, 18]. Liver transplantation is certainly the treatment of choice among patients who have undergone other medical interventions that have been unsuccessful and who have severe liver failure.

## Conclusions

Autoimmune liver diseases are more common in females and young adults. Autoimmune hepatitis and primary biliary sclerosis should be distinguished on the basis of laboratory investigations. AIH can also be associated with vasculitis just like primary biliary sclerosis. It should be treated with immunosuppression, and cirrhosis should be prevented.
